# Renewable energy from biomass surplus resource: potential of power generation from rice straw in Vietnam

**DOI:** 10.1038/s41598-020-80678-3

**Published:** 2021-01-12

**Authors:** Tran Thien Cuong, Hoang Anh Le, Nguyen Manh Khai, Pham Anh Hung, Le Thuy Linh, Nguyen Viet Thanh, Ngo Dang Tri, Nguyen Xuan Huan

**Affiliations:** 1 Faculty of Environmental Sciences, VNU University of Science, Vietnam National University (VNU), 334 Nguyen Trai, Thanh Xuan, Hanoi, Vietnam; 2 Vietnam National Museum of Nature, Vietnam Academy of Science and Technology (VAST), 18 Hoang Quoc Viet, Cau Giay, Hanoi, Vietnam

**Keywords:** Environmental sciences, Bioenergy

## Abstract

Biomass, one of the renewable resources, is expected to play an important role in the world’s energy future. In Asia, rice straw is an abundant agricultural surplus because rice is one of the leading staple food crops in the region. Often, rice straw is burned directly in the field via uncontrolled combustion methods that emit large amounts of short-lived air pollutants, greenhouse gases, and other pollutants. In Vietnam, the energy and environment protection sectors are facing great challenges because of rapid urbanisation and industrialisation. A national strategic choice is to exploit renewable energy, including biomass-derived energy, to achieve energy security and CO_2_ emission reduction. This study investigates the potential of rice straw as an energy source for power plants at a local scale in Vietnam using data derived from satellite Sentinel-1 images. The results show that Vietnam can produce 2,565 MW from rice straw, for which 24 out of 63 provinces have a potential capacity higher than 30 MW, and the Kien Giang province has the highest capacity (245 MW). The study also analyses limitations and obstacles overcoming which can promote the biomass energy sector in the country.

## Introduction

The majority energy resources consumed worldwide are used for power generation, transportation, industry, and community sectors. However, most utility energy derives from fossil oils, gas, and coal. Many countries are struggling to identify alternative energy sources to substitute petroleum and mitigate global warming^1,2^. The use of renewable energy sources (RENS) for power generation is a strategic approach to achieve sustainable development goals (SDG), directly for SDG7, through access to clean, secure, reliable, and affordable energy^2–5^. RENS, is also often called alternative sources of energy, are those resources which can be used to produce energy repeatedly, e.g. biomass energy, solar energy, wind energy, and geothermal energy. The global renewable energy scenarios for year 2030 and 2040 are estimated to have 4289 (34.7%) and 6,351 (47.7%) mill. TOE, respectively^6^. In 2100, the share of RENS is expected to increase very significantly to 30–80%^7^. Among those RENS, biomass resources are considered to have many striking characteristics as it is the only renewable organic resource and one of the most abundant resources worldwide and it can fix CO_2_ in the atmosphere by photosynthesis^8^. Biomass is a group of organic materials which can be divided into wood residues (generated from wood industries); agricultural residues (generated by crops, agro-industries, and animal farms); energy crops (crops and trees intended for energy production); and municipal solid waste^1,5,9–11^. Biomass is the most frequently used RENS, and it provides 3% and 35% of the primary energy in industrialised and developing countries, respectively^10–12^. Approximately 40% of the global population (~ 3.1 billion people) relies on the traditional use of biomass for cooking, especially those living in rural areas of developing countries^3,13^. By 2050, it is estimated that 90% of the world's population may reside in developing countries; thus, biomass energy is likely to remain a substantial energy feedstock^1^. Biomass resources can be used for direct heating in industrial or domestic applications, in the production of steam for electricity generation, or to produce gaseous and liquid fuels^3,10,11^. Direct heating is its most widespread application, but energy production and biofuels are currently gaining considerable interest among energy policy makers.

Rice (*Oryza sativa* L.) is one of the leading staple food crops in Asia, and it generates a significant amount of rice straw after harvest. Rice straw used to be treated in many useful ways such as organic fertilizer, material for cooking, or livestock feeding in the past. These applications have been less popular due to the availability of more comfortable and cheap alternatives. Due to its convenience, open burning of rice straw is done directly in the field few days after harvested^14–16^. This uncontrolled and incomplete combustion method emits large amounts of short-lived air pollutants (e.g. black carbon), greenhouse gases (CO_2_, CH_4_), and other pollutants^16–18^. Besides, burning of rice straw is proved to create loss of nutrients and adversely impacts ecological systems and human health^19,20^. Utility of rice straw as a RENS is promising in solving problem of energy demand and protect the environment at the same time.

In Vietnam, the Resolution No. 55-NQ/TW of the Communist Party Political Bureau establishes specific goals for the energy sector, such as achieving energy self-sufficiency by 2030, and increasing the RENS share of total energy supply to 15–20%. This resolution also aims to produce 320–350 mill. TOE of essential energy by 2045, from which RENS should represent 25–30%. If these goals are met, the reduction of greenhouse gases emitted from the energy sector would be 20% higher compared to a base scenario^21^. As of 2019, the total installed power in Vietnam was 58,880 MW, which represents an increase of 11% compared to 2018^22,23^. From this total capacity, hydropower (12,484 MW, equivalent to 42.2%) and brown coal thermal power (14,595 MW, equivalent to 34.2%) represent the largest portions^23^. The remaining capacity includes oil and diesel thermal power, small-scale hydropower, renewable energy, and imported energy. The total produced power in that year was 240.1 GWh, of which brown coal thermal power shared the largest portion, at 121.5 GWh. Vietnam Electricity Group (EVN) produces 96.25% of the country’s power, other enterprises produce 2.35%, and the rest is imported from China and Laos^22,23^. In 2018, Vietnam has an annual electricity demand increase of 10–15%, which corresponds to a GDP increase from 5.9 to 6.6%. The industrial sector represents 53.6% of the total consumer energy distribution, followed by residential (34.4%), commercial (5.5%), agriculture/forestry (2.3%), and other (4.2%) sectors^4,5,23^. Energy supply sources have been facing many challenges, such as shortage of domestic fossil fuel, fluctuation of oil prices, and decommissioning of a large hydropower dam within the past decade. In this context, exploitation of renewable energy, particularly of biomass energy, can be essential for socio-economic development and environmental security^4^. Vietnam is an agro-industrial country that possesses large amounts of biomass resources, including agricultural wastes that can be used as RENS. In this context, the use of anaerobic digestion for biogas production should be assessed and applied according to the government strategy. Vietnam’s potential for biomass energy from rice straw residue (RISR) is high. However, there is a lack of research to provide sufficient data and information on the potential RISR usability for energy generation. Therefore, this paper investigates the status and potential of RISR to be used as a biomass resource in power plants in Vietnam. Challenges and barriers for the development of RENS biomass are also analysed and discussed.

## Methodology

### Study area

**Figure 1:**
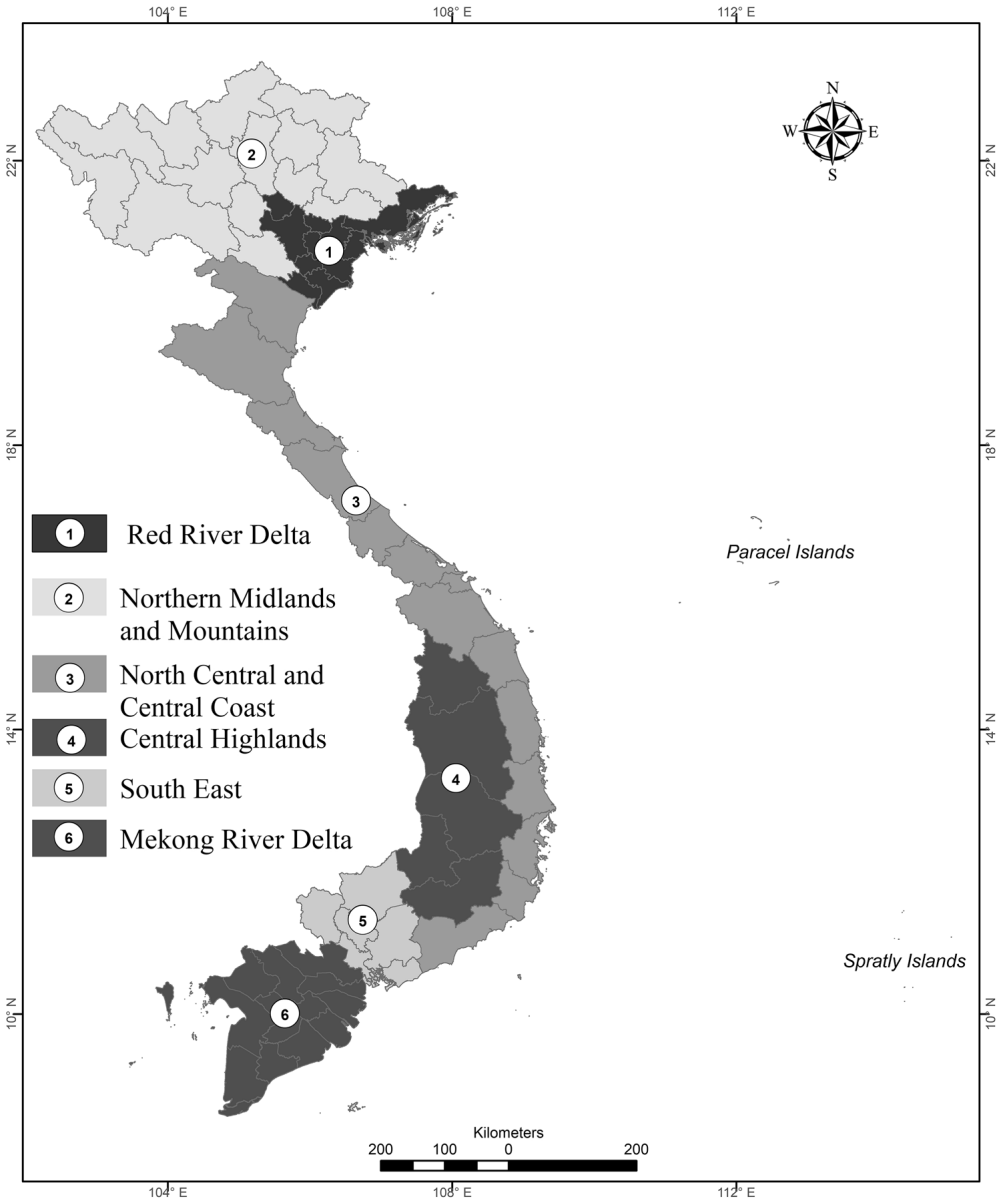
Administrative classification of major socio-economic areas in Vietnam. This map was generated using ArcGIS version 10.2 (https://desktop.arcgis.com/en/arcmap/).

The present study investigated 707 district level localities of Vietnam, including cities under province, urban, and rural districts. These districts belong to 63 provinces with a total population of more than 96 million, which represents a population density of 291 per km^2,24^. The percentage of people living in urban and rural areas are 34.4 and 65.6%, respectively. Five Vietnamese cities have populations over one million, and the two largest are Ho Chi Minh City (population of 8,636,899) and Hanoi (7,781,631 people). The other three cities with over one million people are Hai Phong, Da Nang, and Can Tho. Figure 1 demonstrates the socio-economic regions of Vietnam, where 63 provinces are categorised in six regions.

### Estimation of rice straw surplus in Vietnam

The RISR surplus of Vietnam was established based on information from the satellite Setinel-1A (SS_1A_), as reported by Le et al.^16^. Accordingly, Synthetic Aperture Radar (SAR) SS_1A_ data were incorporated into the ORYZA 2000 crop-growth model^16,25^ to provide more accurate land-cover data by differentiating rice and non-rice land covers based on its rice growing process database. To determine the extent of rice cultivation for the study area over different seasons, time-series SS_1A_ data were acquired based on field knowledge. Using time-series data from land-preparation to harvest periods, we applied our phenology-based classification algorithm to map the rice areas. The conversion from multi-temporal satellite images to mapped rice cultivation areas was performed using MAPscape-RICE, a tool that produces seasonal rice, leaf area index (LAI), and start-of-season (SOS) dates^16,25^. The analysis process showed good agreement with SAR-SS_1A_ statistical data and empirical crop-cut data of Philippines, Vietnam, Cambodia, Thailand, India^25^, and the regional Red River Delta (RRD) of Vietnam^16^. Therefore, the rice cultivated area and rice product for 2019, which were not yet available from traditional data sources of the General Statistics Office of Vietnam (GOSV), were extracted from satellite source data and used to calculate rice straw surplus (RSS).

### Potential rice straw power generation in Vietnam

The RISR production was calculated using a straw to grain ratio (SGR), where the straw residue yield is estimated using input data of rice production^18,26^. SGR varies with season, location, rice species, harvesting method, and cutting height^16,17^. According to Le et al. (2020), a SGR of 1.19 is applicable for Vietnam^16^. Therefore, this value was applied in the present study. The rice straw surplus (RISS), which can be defined as the left-over straw on the field, represents approximately 50% of the RISR on average considering the four seasons in Vietnam^2,14–16^. The loss of RISR during handling and storage was referenced from a similar case in Thailand, at 10%^17^. The overall efficiency (µ) of biomass, including straw power plants, reported in the literature is approximately 20–28%^27,28^, which corresponds well with the efficiency for RISS conversion into energy in Vietnam µ = 25%. The lower heating value (LHV) is 14 MJ kg^−1^^28^. The annual operating hours were assumed to be 8,000 (approximately 330 days)^4,5^. Table 1 summarises relevant parameters adopted for the energy potential estimation of this study. In addition, the potential RISR for energy use in Vietnam is presented in Table 1S supplement data. The power plant production capacity was calculated using equation Eq. (1), which was modified from the referenced Eqs. ^17,29^.

1 $${EP}_{prod}=\frac{RP*SGR*\left(1-LRS\right)*\left(1-MC\right)*LHV* \mu }{3.6*OPH}$$

**Table 1:** Parameters adopted to estimate the rice straw surplus energy potential.

Parameters and indicators		Unit	Value for RSS	Ref.
Straw to grain ratio	SGR	-	1.19	GSOV^24^
Loss of rice straw during handling and storage	LRS	%	10	Nelson^26^
Moisture content assumed on dry basis	MC	%	12	Nelson^26^
Low heating value of RISS	LHV	MJ kg^−1^	14	Koppejan and Van^27^
Foreseen overall efficiency of the plant	µ	%	25	Koppejan and Van^27^
Annual operation hours	OPE	hour	8,000	IOE^4^
Annual RISR on wet basis (wb) in 2019	RISS_wb_	mill. ton_wb_ year^-1^	24	This study
Annual RISR on dry basis (db) in 2019	RISS_db_	mill. ton_db_ year^-1^	21.1	This study

## Results and discussion

### Energy context and distribution of bioenergy in Vietnam

**Figure 2:**
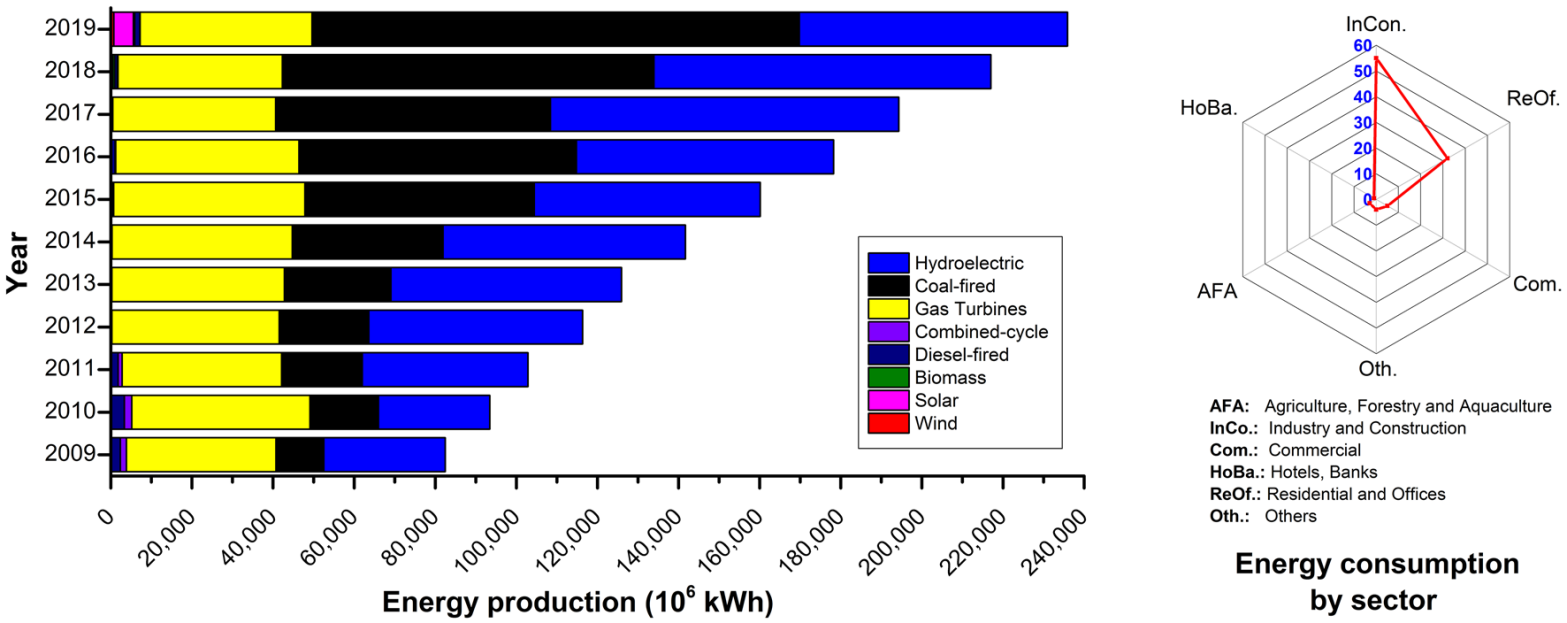
Total energy production from several sources in Vietnam in 2009–2019 *(left),* and energy consumption by sectors in 2019 *(right)*.

Vietnam is endowed with a variety of primary energy resources including coal, diesel, natural gas, and RENS (e.g. solar, wind, biomass, and hydrological), hence it is mostly considered an energy-self-sufficient economy. The total energy production (EN_prod_) in Vietnam in 2009–2019 is shown in Fig. 2. During this period, the average growth rate of total EN_prod_ was 8–15% per year, which was 2–3 times that of the 2001–2011 period, at 4.3% per year^30^. Since 2011, EN_prod_ from coal increased at an annual rate of 10.6–52.2% due to the abundance of coal resources in Vietnam^22^. The EN_prod_ from coal decreased 0.9% from 68,351 GWh in 2016 to 67,714 GWh in 2017, which was a result from the lower coal extraction in 2017. However, the total EN_prod_ increased because EN_prod_ from hydroelectricity increased 35% in 2017 (85,940 GWh) compared to 2016. The most remarkable change was of EN_pro_ from RENS, which increased from 997 MWh in 2018 to 5,890 MWh in 2019, to which solar energy contributed the most with a total production of 4,818 MWh (82%)^22,23^. This increase resulted from the government strategy to promote the RENS sector for green growth and sustainable development goals. In this context, Decision 11/2017/QD-TTg regulates that any power plant project which has been approved by a governmental authority by 30/6/2019 can sell electricity at 9.35 US cent/kWh. As of 2020, Vietnam has not yet issued a tariff for solar electricity but has informed it should be lower than the 9.35 US cent/kWh. The Ministry of Industry and Trade (MOIT) has proposed tariffs of 7.69, 7.09, and 8.38 cent/kWh for lifted, surface, and roof attached solar panels.

In March 2020, the Prime Minister omulgated Decision No. 08/2020/QD-TTg to amend and supplement Decision 24 (24/3/2014) on supportive mechanisms for the development of biomass power plants in Vietnam^31^. Accordingly, the biomass power tariff was significantly adjusted, and the price for biomass-thermal power cogeneration is equivalent to 7.03 US cent/kWh, whereas the price for other biomass power is 8.47 US cent/kWh. Since 2011, the total EN_prod_ in Vietnam has been always higher than the national EN_cons_^22^. However, Vietnam imports/exports electricity from/to neighbouring countries. Electricity is imported from China and Laos for northern provinces, and exported to Cambodia and Laos from southern power plants^22,23^. This was an overall solution to decrease the cost reflective network pricing (CRNP). The total EN_cons_ in 2019 was 238,785 MWh, and the largest energy consumer was the industry and construction sector, which consumed 55.2% of the total EN_cons_, followed by residential and offices (31.8%), commercial (5%), agricultural, forestry and aquaculture (3.1%), and other sectors.

The total biomass energy for power plants was projected to increase from 0.3 mill. TOE in 2015 to 1.8, 9.0, and 20 mill. TOE in 2020, 2030, and 2050, respectively. The respective equivalent power production is expected to increase from 0.6 GWh to 7.8, 37, and 85 GWh. When those are achieved, their respective share considering all power sources in Vietnam should increase from 1% to 3, 6.3, and 8.1%^4^. Vietnam has a largely untapped potential for RENS, and the only significantly used resource is biomass^4,5^.

### Annual surplus rice straw residue in Vietnam

**Figure 3:**
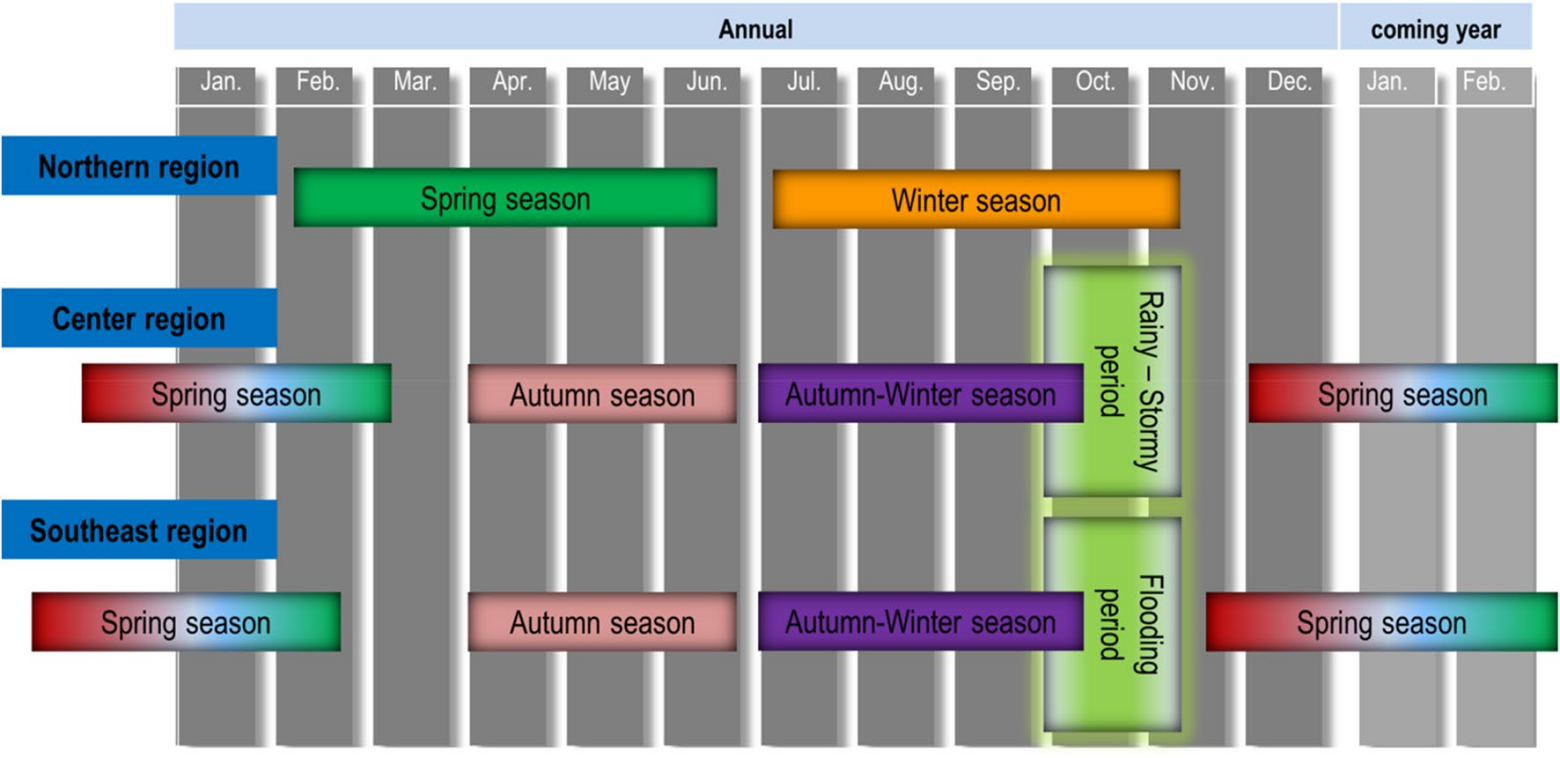
Rice cultivation seasons in three geological regions of Vietnam.

Vietnam has changed its socio-economic region classification over 6 times. At the moment, Vietnam is divided into six administrative areas (Fig. 1), which are numbered from one to six. Rice is cultivated in most areas nationwide. Nevertheless, cultivating calendars vary from 2 to 4 seasons depending on the geological location (northern, central, or southern region)^16,32,33^. Figure 3 illustrates distinguished rice cultivation seasons in the main regions of Vietnam. In the northern regions, due to the subtropical climate, rice is cultivated twice annually, in spring *(Feb.-Jun., Vụ Đông Xuân in Vietnamese)* and winter *(Jul.-Nov., Vụ Mùa in Vietnamese)*. Central and southern provinces have tropical monsoon climate with year-round high temperatures, which leads to one or more cultivation seasons, namely autumn *(Apr.-Jun., Vụ Hè Thu in Vietnamese)* or autumn–winter season *(Jul.-Oct., Vụ Thu Đông in Vietnamese)*. These rice seasons in combination with hydrology, rainfall pattern, and availability of irrigation constitute the variety of rice-based cropping systems^14–16,32,33^. The RRD, North Central and Central coastal (NCCC), and Mekong River Delta (MRD) areas are the major rice production regions in Vietnam, representing 4/5 of the total cultivated land and 85% of the national rice production (Table 2). Therefore, they are the focus of this study.

**Table 2:** Regional distribution and available rice straw residue in Vietnam.

Region	Total area (10^3^ ha)	Rice-planted area (10^3^ ha)	Rice production (10^3^ t)	Rice straw amount (dry 10^3^ t year^-1^)	Potential PPC (MW)
❶	2125.5	1019.6	6179.22	7353.3	354
❷	9520.3	616	3291.27	3916.6	188
❸	9565.5	1321.6	7644.04	9096.4	438
❹	5450.9	296.7	1768.47	2104.5	102
❺	2352	266.9	1388.48	1652.3	80
❻	4081.4	4107.4	24,500.45	29,155.5	1403
Whole country	33,123.6	7628. 1	44,771.93	53,278.6	2565

The total RISR generated in Vietnam was estimated at 54 Mt (Table 2). This amount is significantly higher than those of other countries such as Thailand (32.9 Mt) or Myanmar (34.4 Mt)^33^. The MRD (area 6) accounts for 55% of the total RISR generation and is followed by NCCC (area 3), which accounts for 17% of the total production. The RISR production is high in the two deltas because of the higher rice-planted area and rice yield compared to the other regions.

RISR is one of the most abundant agricultural residues in the world. Mostly, RISR is used for animal feed and soil cover. In Vietnam, most RISR is now left in the fields after harvesting to be burned out in the open^14–16,25,34^. Therefore, RISR has a high potential to partially substitute fossil energy for power generation^1,3,9,17,18,28,29,35,36^. In 2019, rice was cultivated over an area of 7.63 mill. ha in Vietnam, with a total production of 54 Mt, of which more than half (24.5 Mt, 55%) was recorded in the MRD. Kien Giang and An Giang provinces had the largest rice cultivated area, at approximately 700 ha each (Table S1). The NCCC produced 7.65 Mt rice (17%) in 2019. In the RRD region, the highest rice production (1.028 Mt) was recorded in Thai Binh province, and the lowest (0.2 Mt) in Quang Ninh, which is famous for coal mines and coastal zones. In Northern Midlands and Mountain area (NMM), Bac Giang produced the highest amount of rice at 0.6 Mt. The CHA and South East (SE) areas recorded significantly low rice production, at 4 and 3%, respectively. In CHA, most agricultural lands are used for perennial industrial crops (rubber, coffee, and pepper). SE, where Ho Chi Minh City is located, is an area of industrial, commercial, and service zones. Table S1 presents the total rice production in 2019 by province/city level.

**Figure 4:**
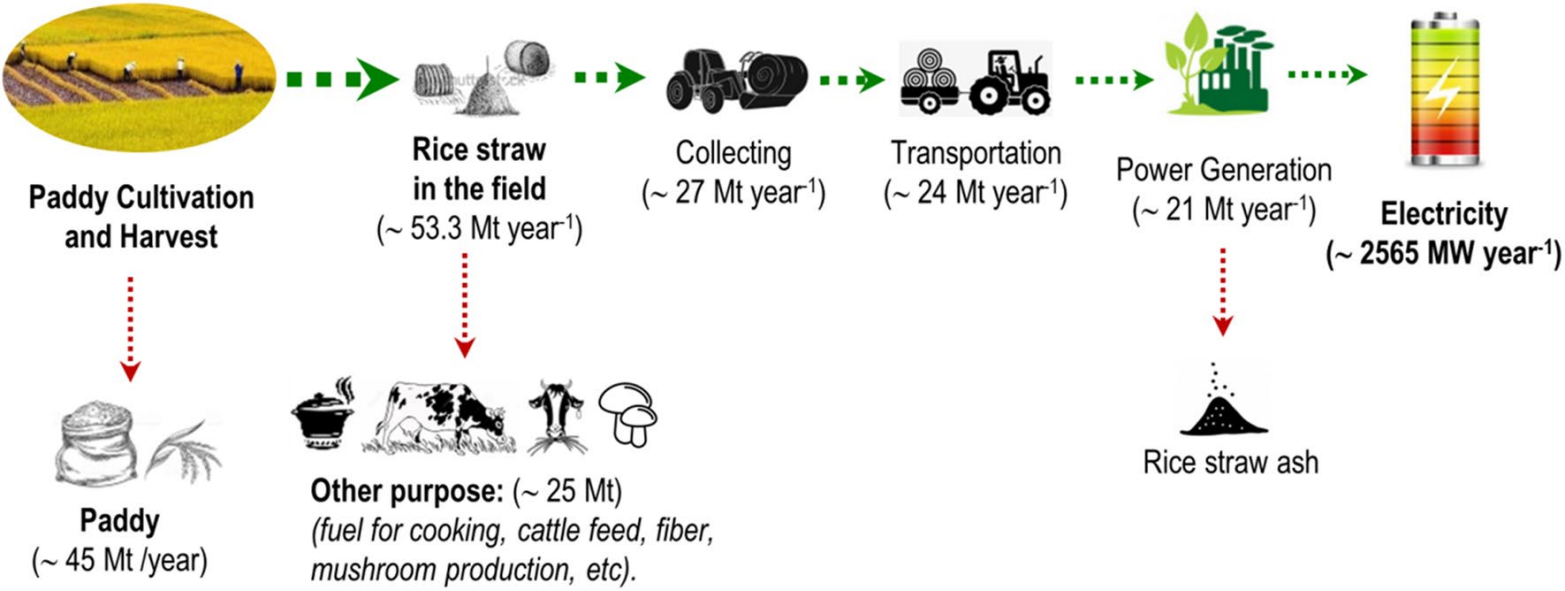
Mechanism and quantification of the potential use of rice straw for electricity generation in Vietnam. This map was generated using ArcGIS version 10.2 (https://desktop.arcgis.com/en/arcmap/).

The rice plant is composed of paddy, straw, and stubble, and only the RISS is used for power generation. Compared with the quick earnings obtained from a paddy crop, the higher investment cost of RISR utilisation provides insufficient incentive for farmers to collect it. Therefore, burning is still the most common practice for RISR disposal. Figure 4 shows an overall evaluation of the average rice straw availability, RISS for electricity generation, and rice straw power plant capacity (PPC) in Vietnam.

### Potential of power generation from rice straw in Vietnam

The potential power generation capacity from RISR for Vietnam is 2,565 MW (Table 2), which represents the lowest share compared to the total production, which is ~ 54,880 MW (2019). However, this amount is significantly larger than the power generated from current renewable sources, i.e. wind and solar, at 135 MW^22^. Table S2 shows the potential capacity of each province. The lowest capacities were obtained at 1.5, 1.6, and 1.8 MW for Tay Ninh, Binh Duong, and Da Nang, respectively, whereas the highest potential capacity was obtained for Kien Giang at 245 MW, followed by Dong Thap and An Giang at 190 and 225 MW, respectively. These three provinces are located in the MRD area and share a boundary with each other. Season-wise, as described in Fig. 3, the three regions of the country have distinct cultivation seasons, of which the spring paddy season is the most suitable period for rice planting, which results in the highest power potential capacity compared to other seasons. Fig. S1-S4 show the distinguished seasonal potential of power generation capacity for each province in Vietnam in 2019.

**Figure 5:**
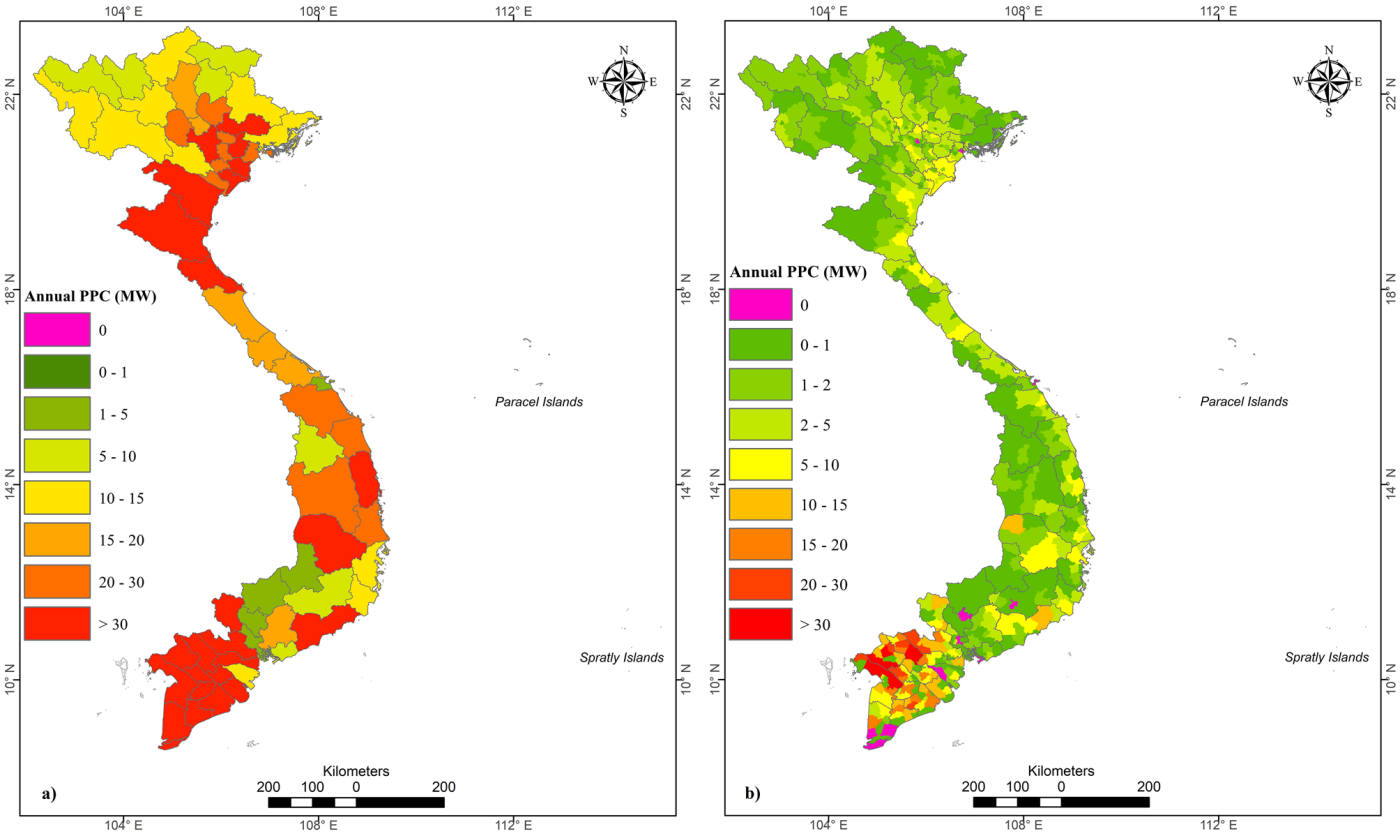
Geographic distribution of power plant capacity based on rice straw at (**a**) provincial and (**b**) district levels in Vietnam, 2019. This map was generated using ArcGIS version 10.2 (https://desktop.arcgis.com/en/arcmap/).

Table 3 categorises the provincial potential electricity generation capacity into five groups. Group 1 includes Binh Duong, Da Nang, Binh Phuoc, and Dak Nong, with a total capacity of less than 5 MW. Group 2 (5–10 MW) comprises seven provinces with a total potential of 53.5 MW, and group 3 (10–20 MW) has 16 provinces, with a total capacity of 227.2 MW. Group 4 has a high RISS potential, and it includes 11 provinces, with total capacity of 257.5 MW. Group 5 includes 24 provinces and has the highest RISS potential for PPC, with a total capacity of 2014.3 MW, which represents 80% of the national biomass electricity capacity. Half the provinces from group 5 belong to the MRD. The geological locations of the provinces’ PPC are shown in Fig. 5.

**Table 3:** Potential of 63 provinces in Vietnam based on RISS availability.

Group no	Identification	Provincial capacity (MW)	No. of Provinces	Group total capacity (MW)	Percentage PPC (%)
1	Very low supply potential	≤ 5	5	12.5	0.5
2	Low supply potential	5–10	7	53.5	2.1
3	Medium supply potential	10–20	16	227.2	8.9
4	High supply potential	20–30	11	257.5	10
5	Very high supply potential	> 30	24	2014.3	78.5
	**Total**		**63**	**2565**	100

### Limitations, barriers, and policy recommendations

Limitations for energy generation from surplus rice straw residue in Vietnam.

#### Other uses of biomass resource

In the traditional rice cultivation, RISR is usually transferred from harvested fields and stored at home as a fuel for cooking or cattle feeding. However, due to the large amounts of waste and modernisation, most farmers burn RISR in an open field. With the improvement of living standards, farmers are using more commercial fuels than agricultural waste products for cooking. Thus, RISR is not as useful as before^2,4,34^, despite its potential value. For example, RISR can be used in cattle feed, compressed boards, and insulation materials for transporting fruits and craft products. It can also be used to produce fertiliser as a way to return nutrients to the soil.

#### Economic feasibility of the required technology

Due to the high price of biomass technology compared with traditional fossil fuel ones, it is not feasible to introduce a new technology in the Vietnam market. In this context, it is important to note that Vietnam has abundant coal sources with low extraction and transportation costs. Moreover, environmental costs are not considered when determining thermal electricity price, which leads to a relatively low price of electricity from traditional sources. For a developing country such as Vietnam, investing in advanced energy technology is not a development strategy priority.

#### Environmental obstacles

Biomass energy can generate certain environmental impacts. In combustion processes, biomass emits PM_2.5_, PM_10_, BC, SO_2_, NO_x_, CO_2_, CH_4_ to the atmosphere. The level of pollution depends on the type of biomass, technology, and mitigation measures. Moreover, large-scale agricultural biomass encourages the development and use of genetically modified species, pesticides, and chemical fertilisers, which can be harmful to natural environments. The production of biomass energy from wood might put a significant pressure on forests. These environmental aspects should be deliberately considered for biomass development plans.

### Barriers

#### Institutional barriers

The Vietnamese government has taken initial steps to encourage the development of renewable energy, including biomass energy. Incentive programmes for investors have been implemented including priority credit, enterprise tax reduction, land rent reduction, and the power purchase agreement (PPA). The MOIT has proposed several other policies such as feed-in tariff for solar, wind, solid waste, and biomass power. Resource wise, Vietnam is considered to have sufficient potential for renewable energy development. However, the proportion over total produced power is still small. The main reasons for this situation are related to institutional issues, such as the lack of a powerful focal point in the government system to coordinate and a national master plan for renewable energy. Consequently, policies are issued in a scattered manner without sufficient investigation data on the potential, demand, and usage of renewable energies. The incentive policies are performed ineffectively, especially for projects in remote and off-grid regions.

#### Technical and technological challenges

Reports of the EVN show that the development of renewable energy, especially biomass power, could be a challenge for electricity operation systems. Renewable power is scattered and seasonal climate-dependent, which may impact the national electricity network. There is a significant lack of data available for renewable energy sources, thus leading to a high uncertainty on potential evaluation. A few investigations on biomass energy resources have been conducted in Vietnam, and they investigate some aspects of the potential for biomass power production. Nevertheless, data from those investigations are quantitatively and qualitatively insufficient for a feasibility assessment. There is a lack of information provision about renewable energy technology. Even though the technology has reached a commercial stage, the information for field application in Vietnam is still lacking.

There are few suppliers of renewable energy equipment and services. The majority of technology is imported, with limited customer and maintenance service, particularly for rural and remote areas where both demand and resources are higher. Because of these imports, domestic human resources present a shortage of experience and skill for compatible equipment choice, operation, and maintenance. These challenges are increasingly severe for biomass energy, which is not well recognised in Vietnam despite the fact that it has been tested and applied in many countries.

In year 2017, Vietnam Institute of Energy (IOE) drafted the national biomass power development plan to 2025 with vision to 2035 in which four main biomass power generation technologies were introduced including steam turbine (ST), gas turbine (GT), internal combustion engine (ICE), and integrated gasification gas engine (IGGE). IOE suggested that the ST appeared to be the most appropriate technology because of its completion in terms of technical aspect which has been commercialized. ST uses input biomass material as solid state and produces high output efficiency that can be applied for 5–10 MW power plants.

#### Financial and economic challenges

In general, renewable energy projects often face the challenge of obtaining capital investment. Two common financial obstacles, namely the lack of approach to appropriate financial resources and lack of a sustainable funding mechanism, are applicable to the renewable energy sector in Vietnam.

Regarding the appropriate financial source, term loans are the main issue. The required investment for renewable energy is significantly higher than that for thermal power, which leads to a dependence on term loans. A typical term loan of 5–8 year is applied in most commercial banks, thus the cash flow for the initial year is minimised. Consequently, the payback period is enlarged, which discourages the contribution of shareholders.

The national strategy for energy development has shown this financial approach limitation for renewable energy. Therefore, a possible solution is the distribution of the Official Development Assistance (ODA) capital and other inter-governmental agreement loans for the development of renewable energy projects.

#### Geographical obstacles

Due to geographical conditions (Fig. 1), Vietnam’s topography has an elongated ***S*** form that lies in the East of the Indochinese Peninsula (middle of Southeast Asia), thus being a long narrow country. Most of Vietnam’s territorial land has mountains and hills concentrated in north and west, whereas plain areas lie mainly in the east and south of the country. The northern provinces are mainly mountainous area, central highlands, and hills; the south is mainly represented by coastal lowlands; and the middle region is relatively flat, along coastal plains, but it is narrow, thereby with low potential (weather conditions) to be a rice granary. Therefore, it is difficult to collect, transport, and store raw materials for electricity production from surplus RISR. Transportation modes that can affect the cost of biomass electricity production should be investigated in future studies.

## Conclusions

The use of biomass as a renewable resource for energy supply in Vietnam and other countries can bring duo benefits as it helps to diminish the pressure of energy demand in one hand, and mitigate environment and climate problems on the other hand. The potential use of rice straw as an input for power plants in Vietnam was studied based on the current RISR availability, limitations, and barriers. The results indicate that the potential for RISR use as a biomass resource for power generation in Vietnam is as high as 2,589 MW for the entire country. The smallest capacities were observed in Binh Duong and Da Nang, at 1.8 MW each, and the highest capacity is in Kien Giang, at 245 MW. Future research should include the environmental and economic effectiveness of RISR power plants in Vietnam.

## Supplementary information

Supplementary Information 1.

## Abbreviations

CHA: Central Highlands areas of Vietnam
EN_coms_: Energy consumption
EN_prod_: Energy production
GOSV: General Statistics Office of Vietnam
LHV: Low heating value of rice straw surplus (MJ kg^-1^)
LRS: Loss of rice straw during handling and storage (%)
NCCC: North central and central coastal areas of Vietnam
NMM: Northern midlands and mountain areas of Vietnam
MC: Moisture content assumed on dry basis (%)
MRD: Mekong River Delta areas of Vietnam
OPE: Annual operation hours (h)
PPC: Power plant capacity (MW)
RENS: Renewable energy sources
RISR: Rice straw residue (ton)
RRD: Red River Delta areas of Vietnam
RISS: Rice straw surplus (ton)
RISS_wb_: Annual rice straw surplus on wet basis (ton year^-1^)
RISS_db_: Annual rice straw surplus on dry basis (ton year^-1^)
SEA: South East areas
SS_1A_: Satellite Setinel-1A
TOE: Tonne of oil equivalent (ton

## Greek letters

µ: Foreseen overall efficiency of the plant (%)
