# Supplementary material for: Renewable energy from biomass surplus resource: potential of power generation from rice straw in Vietnam
Source: Sci Rep. 2021 Jan 12;11:792. doi: 10.1038/s41598-020-80678-3 (PMC7804265; doi:10.1038/s41598-020-80678-3)
Supplement: Supplementary file 1 — Supplementary Information 1. [file 41598_2020_80678_MOESM1_ESM.docx]

**Table S1.**

The datasheet of rice planted area (ha), rice production (ton) in Vietnam 2019


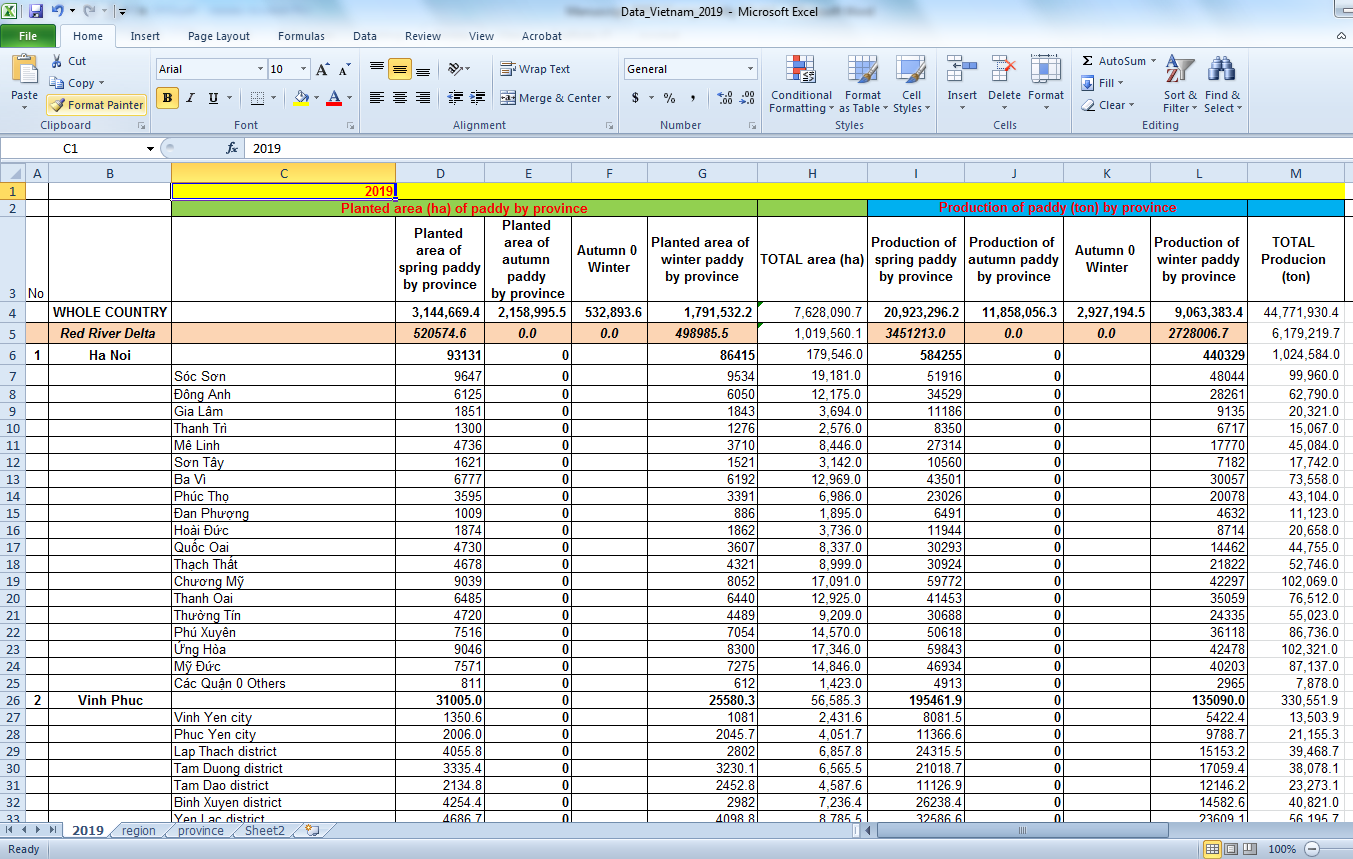


**Table S2.**

The datasheet of available rice straw residue (RISR), rice straw surplus (RISS), of rice planted area (ha), rice production (ton) for electric generation in Vietnam 2019.


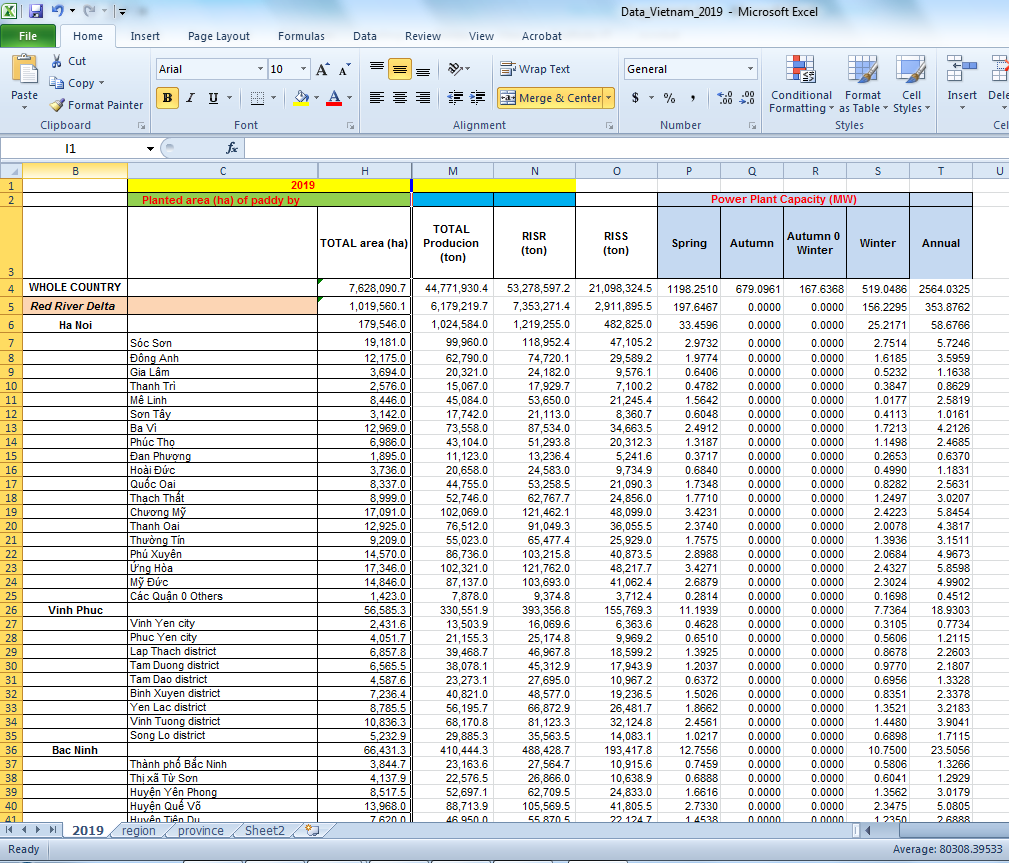


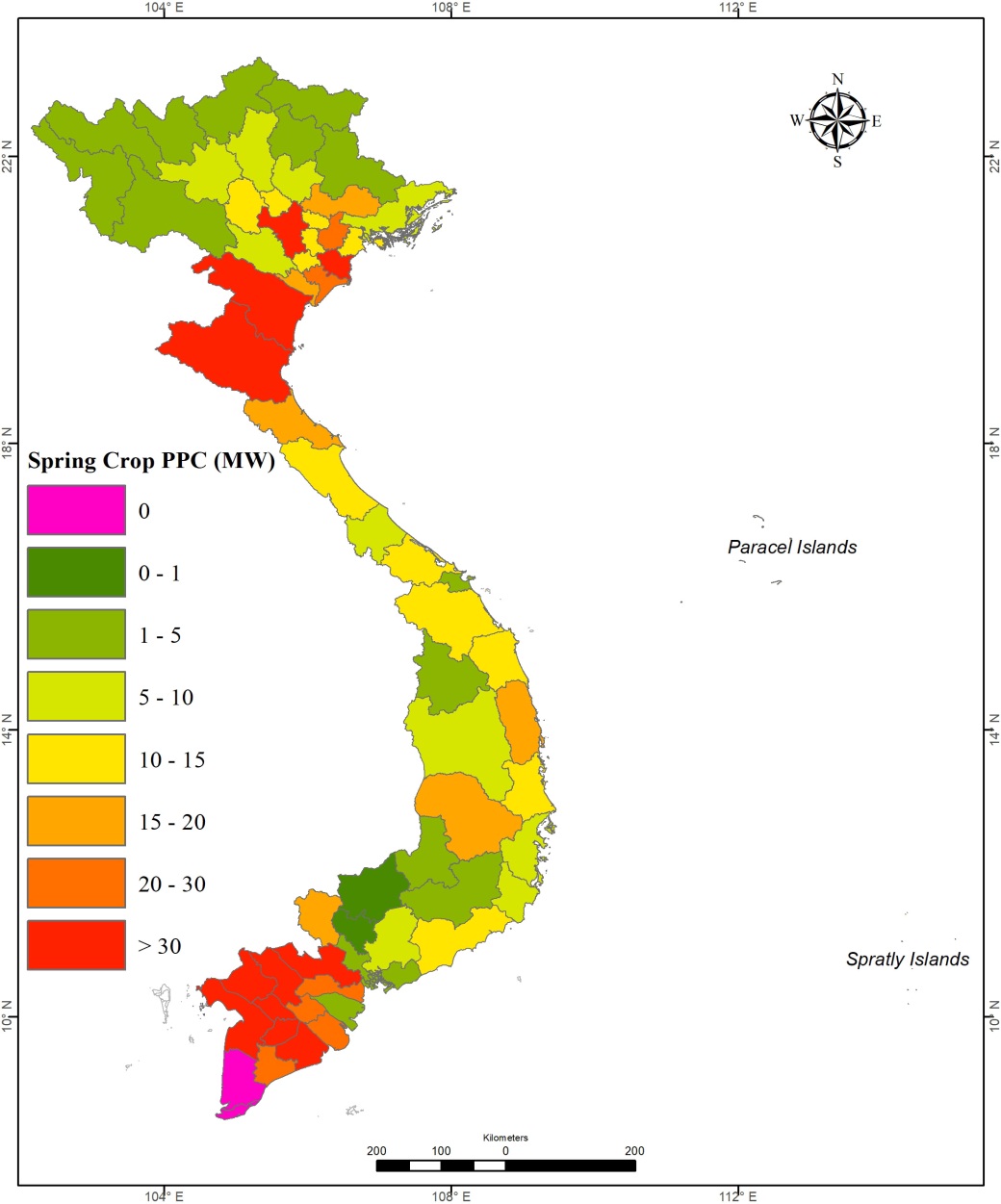


**Fig. S1.** Geographic distribution of power plant capacity based on rice straw at provincial levels in Vietnam, spring season 2019. This figure was generated using ArcGIS version 10.2 (<https://desktop.arcgis.com/en/arcmap/>).


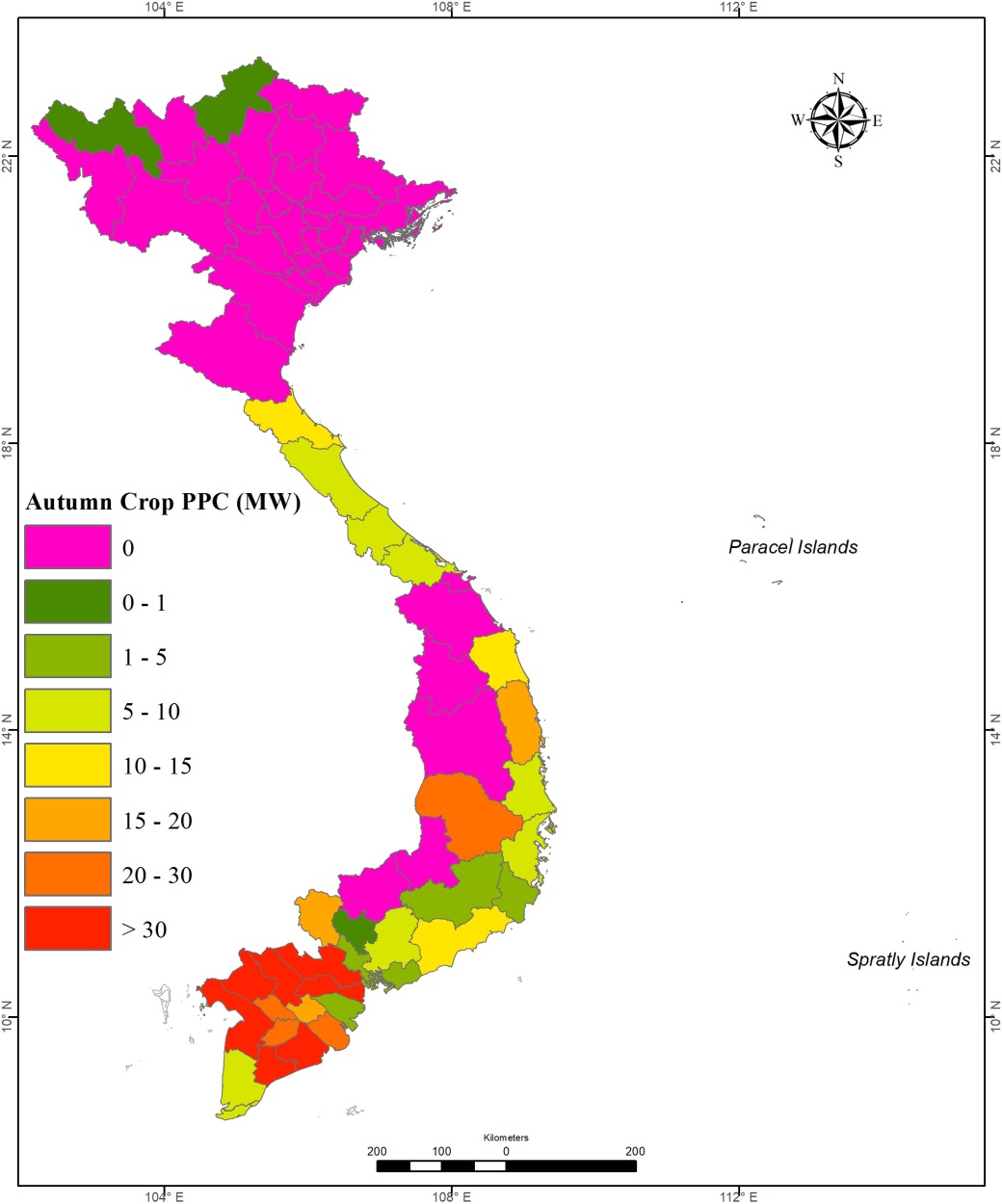


**Fig. S2.** Geographic distribution of power plant capacity based on rice straw at provincial levels in Vietnam, autumn season 2019. This figure was generated using ArcGIS version 10.2 (<https://desktop.arcgis.com/en/arcmap/>).


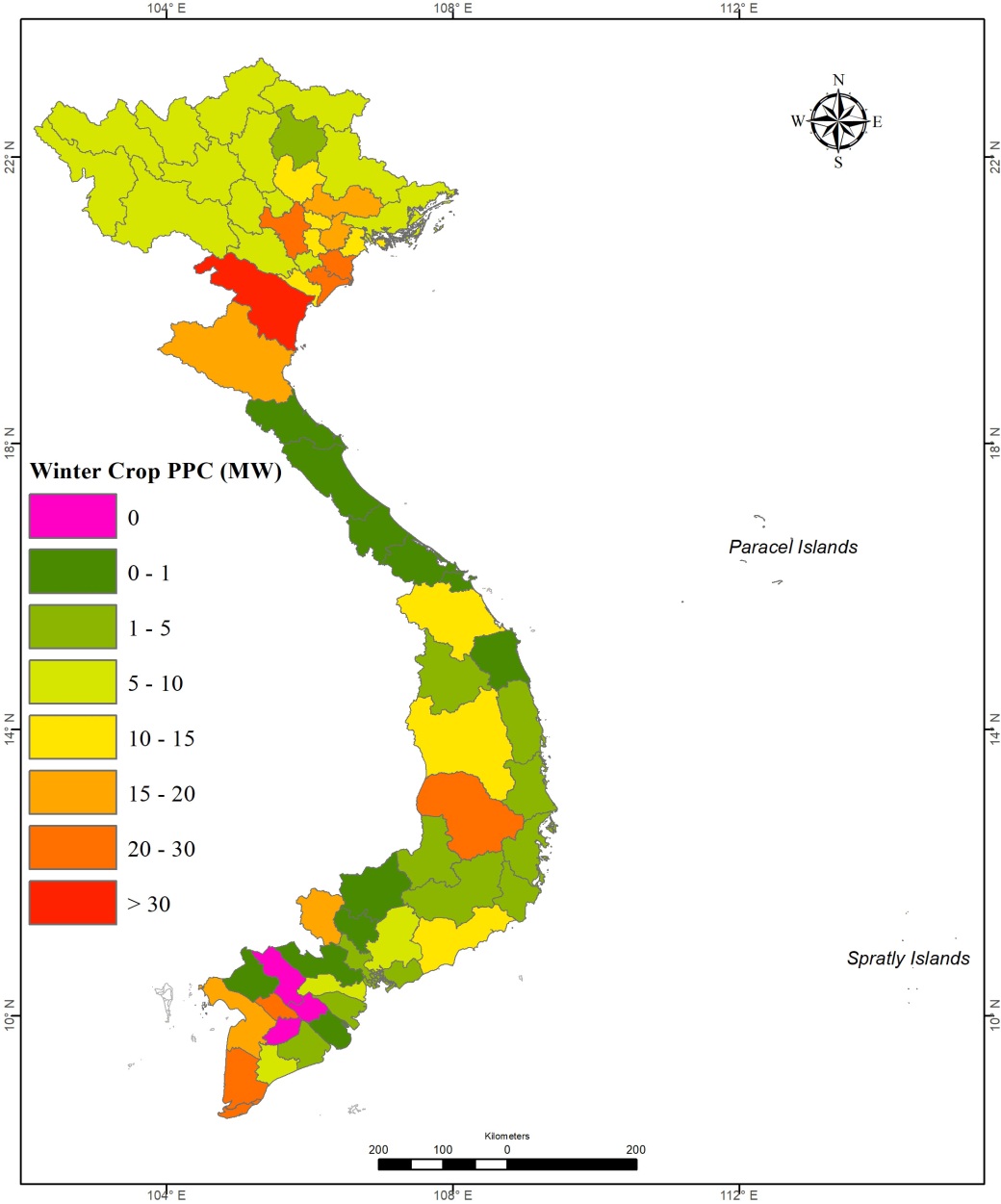


**Fig. S3.** Geographic distribution of power plant capacity based on rice straw at provincial levels in Vietnam, winter season 2019. This figure was generated using ArcGIS version 10.2 (<https://desktop.arcgis.com/en/arcmap/>).


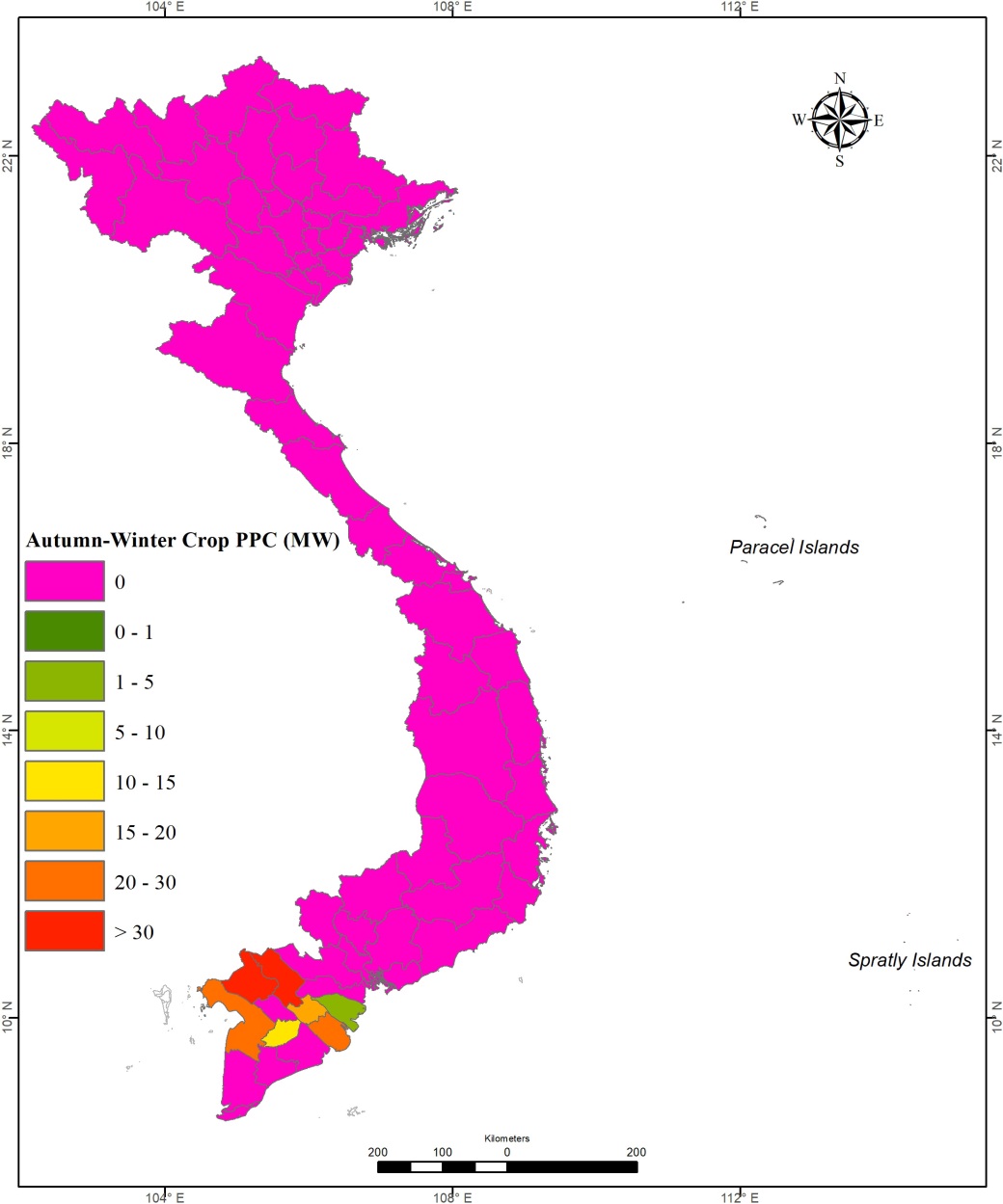


**Fig. S4.** Geographic distribution of power plant capacity based on rice straw at provincial levels in Vietnam, autumn-winter season 2019. This figure was generated using ArcGIS version 10.2 (<https://desktop.arcgis.com/en/arcmap/>).
